# Single-Atom Platinum Catalyst for Efficient CO_2_ Conversion via Reverse Water Gas Shift Reaction

**DOI:** 10.3390/molecules28186630

**Published:** 2023-09-14

**Authors:** Yulian He, Dahong Huang

**Affiliations:** 1 University of Michigan and Shanghai Jiao Tong University Joint Institute, Shanghai Jiao Tong University, Shanghai 200240, China; yulian.he@sjtu.edu.cn; 2 Department of Environmental Science and Engineering, University of Science and Technology of China, Hefei 230026, China

**Keywords:** single-atom catalysts (SACs), CO_2_ reduction, reverse water gas shift (RWGS), 100% selectivity, thermal stability

## Abstract

The need to tackle CO_2_ emissions arising from the continuously rising combustion of fossil fuels has sparked considerable interest in investigating the reverse water gas shift (RWGS) reaction. This reaction holds great promise as an alternative technique for the conversion and utilization of CO_2_. In this study, a scalable method was employed to synthesize a single-atom Pt catalyst, uniformly dispersed on SiC, where up to 6.4 wt% Pt_1_ was loaded onto a support based on ligand modification and UV photoreduction. This Pt_1_/SiC catalyst exhibited a high selectivity (100%) towards the RWGS reaction; 54% CO_2_ conversion was observed at 900 °C with a H_2_/CO_2_ feed-in ratio of 1:1, significantly higher than the conventional Pt nanoparticle counterparts. Moreover, Pt_1_/SiC displayed a robust stability during the long-term test. The activation energy with as-synthesized Pt_1_/SiC was further calculated to be 61.6 ± 6.4 kJ/mol, which is much lower than the 91.6 ± 15.9 kJ/mol of the Pt nanoparticle counterpart and other Pt-based catalysts reported so far. This work offers new insights into the utilization of diverse single-atom catalysts for the RWGS reaction and other crucial catalytic processes, paving the way for the further exploration and application of SACs in various industrial endeavors.

## 1. Introduction

Noble metals have been extensively explored as catalysts and co-catalysts, since they exhibit exceptional performance in catalyzing various reactions such as hydrogen evolution [1,2], hydrogenation [3], and alcohol oxidation [4,5]. However, the scarcity of noble metals in nature and the resultant high costs have restricted their potential in large-scale commercial applications [6,7]. The last decade has witnessed a rapid development on single-atom catalysts (SACs), which have attracted extensive attention due to the superior catalytic performance in various important processes [8]. The most important advantage of SACs, compared to their nanoparticle counterparts, is the maximized atom efficiency (~100%), where each atom is accessible to the reaction due to atomic dispersion [9]. Previous studies have identified additional benefits, such as a strong interaction with the supportive substrate, the absence of metallic bonds, and a low coordination state [10,11]. In particular, the low coordination state of metal is considered critical for the improved catalytic performance of SACs, compared to their metal oxide counterparts, which also lack metallic bonds [12]. SACs represent the forefront of catalysis research, and offer ideal models to understand catalytic behavior at the atomic level, addressing the emerging concerns over the design and optimization of catalysts that remains a fundamental challenge and is of great interest in regard to the practical applications.

The ever-growing concentration of carbon dioxide (CO_2_) in the atmosphere, and its detrimental impact on climate change, have spurred widespread research efforts aimed at developing effective strategies for CO_2_ reduction. CO_2_ conversion and utilization, aligning with the “waste-to-wealth” concept through the prism of green chemistry, have gained significant attention as feasible approaches to mitigate greenhouse gas emissions while simultaneously producing valuable chemicals and fuels. Among the alternative processes, the reverse water gas shift reaction (RWGS, CO_2_ + H_2_ → CO + H_2_O) is garnering immense attention as one of the most promising routes for sustainable and efficient carbon management [13,14]. CO, as the simplest CO_2_-derived product, can be further exploited as a building block for various value-added hydrocarbons via Fischer–Tropsch synthesis [15,16]. Moreover, RWGS serves as a crucial intermediate step in many other hydrogenation processes, such as the Sabatier reaction [17] and methanol synthesis [18], fostering the development of a more circular and sustainable economy and contributing to the transition to a low-carbon future. Due to the vital importance of RWGS reactions, the rational design of catalysts has thus attracted global attentions.

A well-designed catalyst for the RWGS reaction should possess several critical characteristics. First, high selectivity is important in order to minimize undesired reactions and to avoid the formation of byproducts, maximizing the efficiency of the target reaction and eliminating the need for downstream separation processes. Second, the impressive activity is also critical to enable the reaction at relatively low temperatures by reducing the energy barrier. These two features improve the overall efficiency of the conversion of CO_2_ and H_2_ to CO and H_2_O, respectively. Furthermore, it is imperative that the catalyst demonstrates robust stability under the operational conditions, retaining structural integrity and sustained catalytic performance throughout the challenging scenarios of elevated temperatures and the resultant corrosion. Pt-based catalysts have been regarded as promising candidates in meeting the aforementioned requirements based on reported studies [19,20]. However, the scarcity of Pt in nature leads to elevated costs, which imposes constraints on practical applications. To address this challenge, the emergence of SACs offers a potential solution by maximizing the atomic efficiency of Pt-based catalysts. In SACs, each Pt atom participates in the reaction as an active site on the catalyst’s interface, reducing the Pt loading and overall cost. Additionally, the presence of strong metal–support interactions in SACs can guarantee the stable coordination structure of single-atom Pt catalysts, enhancing their longevity and performance over extended periods. However, most SACs are limited by their low loading amount, which is typically below 1%. Such low loading quantities are necessary to prevent the aggregation of atomically dispersed metal centers into nanoparticle counterparts during the synthesis and reaction. Unfortunately, the limited number of active sites resulting from low loading negatively impacts the overall activity of SACs. Therefore, developing single-atom Pt catalysts with a high loading amount is essential to meet the urgent demand for practical implementation.

In this work, we introduce a simple method to load a 6.4 wt% Pt catalyst on a silicon carbide (SiC) substrate, utilizing a 254 nm UVC photoreduction method. We exploit a wide range of techniques, including X-ray absorption fine structure spectroscopy (XAFS), aberration-corrected high-angle annular dark-field scanning transmission electron microscopy (AC-HAADF-STEM), and X-ray photoelectron spectroscopy (XPS), to fully characterize the synthesized nanostructures. The catalytic performance of atomically dispersed Pt surpasses that of conventional Pt nanoparticle catalysts. This superiority can be attributed to two factors: the near 100% atomic utilization of Pt_1_, and the inherent higher catalytic performance of Pt_1_ compared to Pt atoms within Pt nanoparticles. The exploration of RWGS using the Pt_1_/SiC catalyst holds the potential to deepen our understanding of the catalytic behavior of SACs and broaden their applications in the realm of industrial catalysis, serving as an inspiration for future studies that aim to advance SACs-related research.

## 2. Results and Discussion

**Figure 1:**
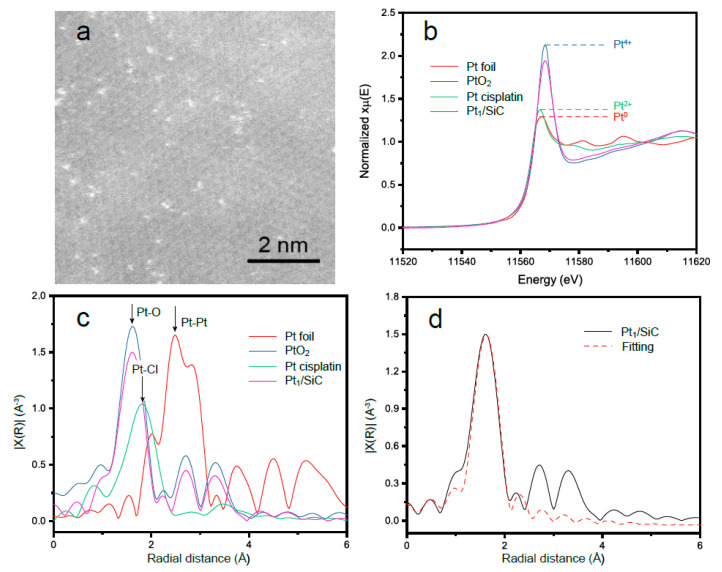
(**a**) AC-HAADF-STEM of Pt_1_/SiC, showing the atomically dispersed Pt single atoms on the SiC substrate; (**b**) normalized XANES measurements at the Pt L_3_-edge of Pt_1_/SiC sample and Pt foil, PtO_2_, Pt cisplatin standards; (**c**) the Pt k^3^-weighted FT-EXAFS of standards and Pt_1_/SiC; and(**d**) the fitting results for Pt_1_/SiC (the parameters extracted from the fit are provided in Table 1). The data ranges used for data fitting in K-space and R-space were 3.0−10 Å^−1^ and 1.2−2.5 Å, respectively.

The atomic dispersion of Pt was confirmed by AC-HAADF-STEM. The estimated radius of the Pt species was approximately 2 Å (Figure 1a), suggesting that the Pt was dispersed at the atomic level rather than forming metallic Pt clusters. Furthermore, the coordination environment of Pt_1_/SiC was investigated by XAFS. The white line (WL) intensity of Pt_1_/SiC from the normalized near-edge X-ray absorption spectroscopy (XANES) analysis, as shown in Figure 1b, qualitatively demonstrated the presence of positively charged Pt atoms between Pt^2+^ and Pt^4+^, indicating the 5*d*-orbitals of the Pt in Pt_1_/SiC are partially unoccupied. The Fourier-transformed extended XAFS (FT-EXAFS, Figure 1c,d) spectra of Pt L_3_-edge indicated that Pt_1_ only bonds with the O atoms, wherein the interatomic distance of Pt-O path in the first coordination shell was determined to be 1.6 Å. The absence of Pt-Pt coordination excludes the formation of Pt nanoparticle, consistent with the AC-HAADF-STEM result. Comparison with the Pt cisplatin reference confirms the disappearance of Pt-Cl bonds in Pt_1_/SiC, suggesting the complete reduction of the PtCl_6_^2−^ precursor. Best-fit parameters extracted from FT-EXAFS data are presented in Table 1. The average Pt-O coordination number equals to 5, implying the unsaturated coordination of Pt atoms in the Pt_1_/SiC, which benefits catalytic performance.

**Figure 2:**
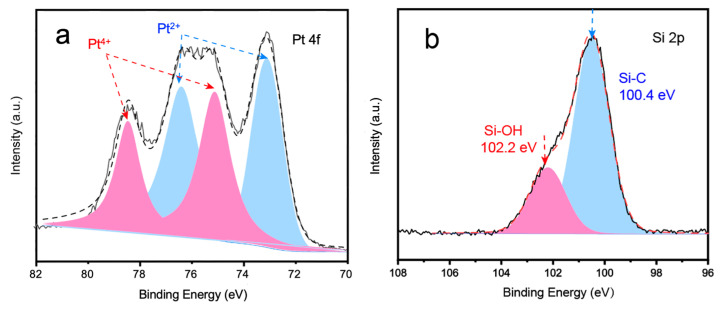
Binding energy of (**a**) Pt 4f Pt_1_/SiC and (**b**) Si 2p for pristine SiC by high-resolution XPS spectra. Pt peak position indicates that the oxidation state of Pt_1_ was between 2+ and 4+, consistent with XAFS results. Si peaks at 102.2 eV indicate surface -OH groups, which are critical to the stabilization of Pt_1_.

The XPS analysis, presented in Figure 2a, quantitatively determined the oxidation state of Pt_1_ to be ~2.9 (55% Pt^2+^ and 45% Pt^4+^), which agrees well with the XANES analysis shown above. The adjacent O atoms originate from the surface -OH groups on SiC [21,22], as evidenced by peaks at 102.2 eV and 532.6 eV from the Si 2p (Figure 2b) and O 1s (Figure S1) regions, respectively. The atomic ratio of oxygen is calculated to be 21.8% by integrating peak area; peak intensities are normalized to the strongest peak. Sufficient -OH groups are of vital importance to the stabilization of Pt_1_, where each Pt atom is stabilized onto the SiC support by coordinating with neighboring oxygen atoms on its surface based on our previous work [3].

**Figure 3:**
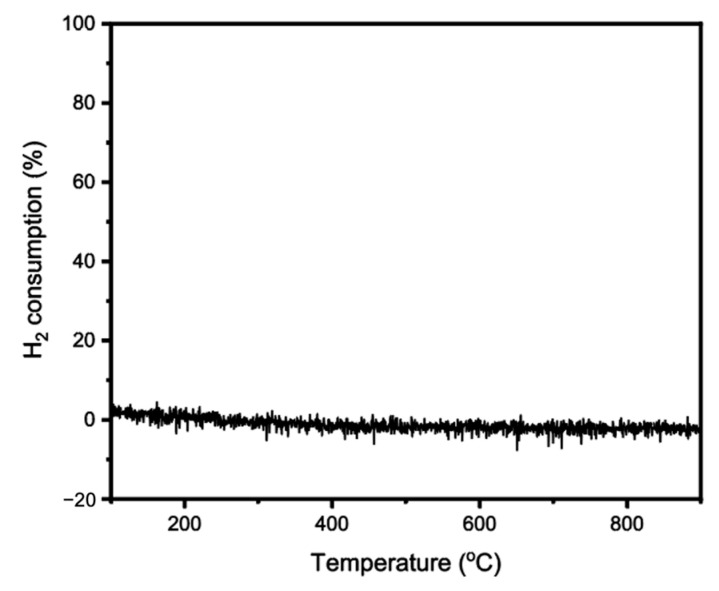
On-line mass spectrometry measurement during TPR process of the Pt_1_/SiC catalyst, the weight hourly space velocity of H_2_ was set at 12,000 mL·g_cat_^−1^·h^−1^.

Prior to the measurement of the actual RWGS reaction, the Pt_1_/SiC catalyst was pre-treated in a H_2_ atmosphere, cleaning the -OH groups from the chemisorbed water that had accumulated on their surfaces [23]. A temperature-programmed reduction (TPR) in 10% H_2_/Ar was conducted to evaluate the pre-treatment conditions. From the recorded mass spectra in Figure 3, we observed no dips in the H_2_ signal up to 900 °C, demonstrating that no H_2_ was consumed during the TPR process. It is therefore deduced that Pt_1_/SiC can retain a remarkable anti-reduction stability even under high temperatures and a reductive atmosphere. To avoid the potential catalyst sintering at high temperatures, the H_2_ pre-treatment was set to 200 °C for 1 h before the RWGS reaction.

**Figure 4:**
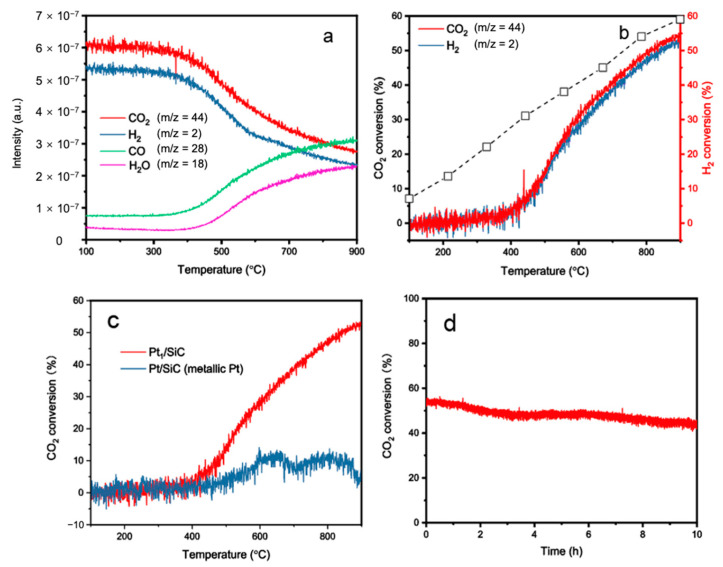
(**a**) On-line mass spectrometry measurements during RWGS reaction of the H_2_ pre-treated Pt catalyst; (**b**) conversion dependence over temperature plot. %CO_2_ conversion and %H_2_ conversion are computed using differences in the *m*/*z* = 44 and *m*/*z* = 2 channels as a function of temperature. The dash line with square corresponds to the theoretical equilibrium conversion under the H_2_/CO_2_ = 1:1 feed-in condition; (**c**) CO_2_ conversion for single atom Pt catalyst and conventional Pt nanoparticles as a function of temperature; (**d**) long-term stability test of Pt_1_/SiC at 900 °C.

The RWGS reactions were conducted in a flow reactor setup, as illustrated in the SI (Figure S2). The full mass spectrometry and conversion dependence over temperature plots are given in Figure 4a,b, respectively. Using the Equation (1) below, CO_2_/H_2_ conversion was tested at each temperature point. Measurements were taken approximately every 3 s. No change in the background argon signal was observed in the mass spectra, suggesting that the partial pressure in the chamber remained unchanged over the reaction course.

(1) $$\%{CO}_{2}(H_{2})\;Conversion=(1-\frac{Measured\;{CO}_{2}(H_{2})signal}{Pre-reaction\;{CO}_{2}(H_{2})signal})\times 100\%$$

According to Figure 4b, it can be concluded that the RWGS reaction is endothermic, which is supported by the observation that the reaction is more favorable at higher temperatures, consistent with the findings previously reported in the literature [24]. Mass spectrometry analysis revealed that no hydrocarbons other than CO (e.g., CH_4_, C_2_H_6_, etc.) were detected, indicating a 100% selectivity of CO_2_ to CO. This high selectivity at low temperatures (<700 °C) is valuable, as the consumption of H_2_ feed can be significantly lowered by the suppression of other competing H_2_-intensive pathways such as methanation [25]. Moreover, the onset temperature of the reaction was approximately 280 °C (Figure 4b), falling within the range of many other reported values for Pt-based catalysts. The maximal CO_2_ conversion was found to be around 54% at 900 °C, corresponding with a turnover frequency (TOF) of 0.49 mol_CO2_ mol_Pt_^−1^ s^−1^ (Section S1). This represents a significant improvement compared to other studies on supported Pt catalysts for the RWGS reaction, approaching the thermodynamic equilibrium conversion under the H_2_/CO_2_ = 1:1 feed-in conditions [26]. Due to the endothermic nature of the RWGS reaction, very limited catalysts reported so far have exhibited a conversion rate of more than half (50%) at above 800 °C. Some exceptions include NiO supported on SBA-15, with 55% conversion of CO_2_ at 900 °C and 100% selectivity to CO [27], 1D hematite nanowire with 55% conversion of CO_2_ at 850 °C and 100% selectivity to CO [24], and 2D MnO_2_ nanosheets with 50% conversion at 850 °C [25]. A comparative table regarding important kinetic parameters of various typical catalysts for RWGS is concluded in Table S1.

To serve as a control, a conventional Pt nanoparticle counterpart supported by the SiC substrate (prepared using the hydrothermal method, Figure S3) was tested under the same experimental procedures, as shown in Figure 4c. The results show that the conventional Pt nanoparticle catalyst exhibited significantly lower catalytic performance in converting CO_2_ to CO, despite having a one-order-of-magnitude higher Pt mass loading, as determined by the ICP-MS results (6.4 wt% of Pt_1_ and 10.3 wt% of metallic Pt, Figure S3). Moreover, the regular Pt/SiC catalyst exhibited a sharp decrease in CO_2_ conversion at 700 °C, and the reaction almost ceased at 900 °C, likely due to the catalyst sintering, caused by surface atom rearrangements and the collapse of the Pt nanostructures and/or poisoning of Pt by CO.

Contrastingly, the Pt_1_/SiC catalyst did not display a comprised activity, with the CO_2_ conversion increasing almost linearly with the reaction temperature, as shown in Figure 4c. The high thermal stability of Pt_1_/SiC can be ascribed to the strong metal–support interaction through interfacial bonds (i.e., Pt-O in this work) between Pt_1_ and the SiC support [28,29]. The superior catalytic performance of the Pt_1_/SiC is probably a result of the promoted CO_2_ adsorption on Pt, stemming from the partially unoccupied 5*d* orbitals, given that the SiC support is highly irreducible itself. Additionally, H_2_ adsorption was found to be unfavorable during TPR, as discussed in Figure 3. Therefore, it is demonstrated that the reaction primarily proceeds through CO_2_ adsorption on Pt first, presumably through the surface Pt-OH species to form surface-bound CO_2_ (mostly in the form of carbonates [30,31]), followed by H_2_ reduction to the final product of CO. Such a reaction pathway has been observed in several Pt-based systems, as well as oxides such as MnO_2_ [25].

**Figure 5:**
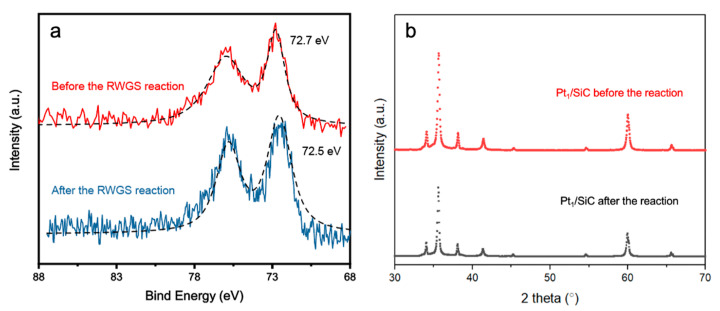
(**a**) X-ray photoelectron spectroscopy (XPS) and (**b**) X-ray diffraction (XRD) analyses of Pt_1_/SiC samples before and after the RWGS reaction, showing Pt 4f peak shifts of XPS spectra.

Exploring the deactivation behavior of a catalyst is crucial, particularly when the catalyst is prone to being poisoned during the reaction course. Pt catalysts are well known to be susceptible to CO poisoning because of the high desorption energy of CO. Therefore, it is essential to assess the stability of the Pt_1_/SiC catalyst under the reaction conditions over an extended period. In this study, 50 mg of catalyst were tested using the same setup discussed above for a duration of 10 h at 900 °C, as shown in Figure 4d. During the first few hours, the Pt_1_/SiC catalyst exhibited excellent stability with negligible activity drop. However, as time progressed, Pt_1_/SiC experienced gradual deactivation. Nonetheless, the CO_2_ conversion still remained relatively stable at approximately 50%, even after 10 h. The slight decline in activity can be attributed to two factors: (1) catalyst poisoning resulted from the occupation of Pt_1_/SiC by CO molecules; and (2) a small amount of Pt_1_/SiC reduction, as demonstrated by the binding energy shift to 72.5 eV, lower than the fresh Pt_1_/SiC catalyst (72.7 eV), as demonstrated by the XPS results in Figure 5a. The change in binding energy (BE) is as follows: Pt foil (71.3 eV) < Pt_1_/SiC after reaction (72.5 eV) < Pt_1_/SiC before reaction (72.7 eV) < PtO_2_ (77.7 eV) [32]. Although the precise regulation of Pt_1_/SiC significantly improves the activity, high temperature leads to instability after long-term use, reflected by a peak shift towards the lower value of 0.2 eV detected in above figure. Though a small amount of Pt_1_/SiC may migrate and aggregate into Pt nanoparticles after the reaction, we would like to point out that Pt_1_/SiC should remain mostly stable. Otherwise, the peak of Pt_1_/SiC should move even closer to that of Pt foil. The negligible difference indicates the high stability of the Pt_1_/SiC catalyst, as shown in Figure 5b. This additionally excludes the existence of Pt nanoparticle due to the absence of corresponding Pt peaks. Further developments are necessary to overcome this essential obstacle, in order to prolong the catalyst’s lifespan to meet the needs of practical applications. In conclusion, our results call for further efforts to design and optimize atomically dispersed Pt-based catalysts to mitigate the catalyst poisoning and further enhance the stability of SACs, extending catalyst’s lifespan for practical applications.

In addition, we also performed a kinetic analysis to compare the apparent activation energies of the two Pt/SiC systems. Based on the principles of collision theory, molecules involved in a chemical reaction must possess sufficient energy to undergo rearrangement and reach the activated states necessary for the reaction to take place. The activation energy, in this context, refers to the minimum energy threshold required for a reaction to occur. Here, we employed the Arrhenius equation (see Section S2) to analyze the catalytic performance of the Pt_1_/SiC catalyst, which provided valuable information for understanding and optimizing the catalytic behavior of the Pt_1_/SiC catalyst.

**Figure 6:**
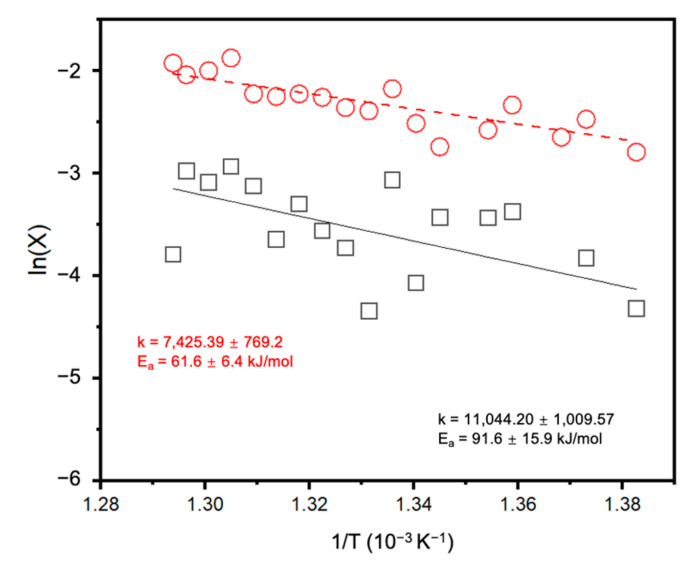
Arrhenius plot for the calculation of apparent activation energy under kinetic control. The red line represents the Pt_1_/SiC; the black one represents the metallic Pt/SiC.

Continuous data points were collected within the range of 450 °C to 500 °C under kinetic control, ensuring CO_2_ conversions below 10% (Figure 6). By performing linear fitting on the data and extrapolating the slope, the apparent activation energy of the Pt_1_/SiC catalyst for the rate reaction of the RWGS process was determined to be 61.6 ± 6.4 kJ/mol. Notably, this activation energy is significantly lower than the value of 91.6 ± 15.9 kJ/mol obtained for the Pt nanoparticle counterpart. It is important to mention that this low activation energy is unusual compared to other reported values in the literature for the RWGS reaction [33]. The observed difference in activation energy between the Pt_1_/SiC catalyst and the Pt nanoparticle catalyst indicates a distinct reaction pathway, i.e., CO_2_ adsorption, H_2_ activation, CO desorption, and so on, which originated from the different properties of atomically dispersed Pt sites. Further investigation is warranted to gain a comprehensive understanding of these underlying factors and to elucidate the specific characteristics that enable SACs to exhibit this superior catalytic performance in the RWGS reaction.

## 3. Materials and Methods

### 3.1. Reagents and Materials

All chemicals used in this work were of reagent grade or higher and were used as-received without further purification. Aminopropyltrimethoxysilane (APTMS), platinum oxide (PtO_2_), and cis-diammineplatinumdichloride (Pt(NH_3_)_2_Cl_2_, Pt cisplatin) were obtained from Sigma-Aldrich, Burlington, MA, USA. Silicon carbide (beta-phase, nanopowder, 95% purity) was purchased from Alfa Aesar, Ward Hill, MA, USA. Chloroplatinic acid (H_2_PtCl_6_, 8 wt% in H_2_O) was obtained from J&K Scientific (San Jose, CA, USA). Hexane was supplied by J. T. Baker, Phillipsburg, NJ, USA. All aqueous solutions were prepared with ultrapure/deionized water obtained from a Milli-Q system, Bay City, MI, USA.

### 3.2. Preparation of Pt_1_/SiC Catalysts

The Pt_1_/SiC was synthesized by photoreduction of H_2_PtCl_6_ into Pt_1_. As shown in Figure S4, the potential of zero charge (PZC) of pristine SiC was estimated to be 9 according to the zeta-potential measurement. Thus, we dispersed SiC into the deionized (DI) water solution using sonication with a pH of 4, mediated by the H_2_SO_4_, where the surface of SiC would be highly positively charged. Subsequently, an electrostatic interaction took place between the negatively charged Pt precursors (PtCl_6_^2−^) and the positively charged SiC, ensuring good distribution of Pt precursors onto the surface of SiC. Pt precursors were photo-reduced under a moderate ultraviolet C (UV-C) irradiation of 7.8 mWcm^−2^. The synthesized Pt_1_/SiC was finally separated by centrifugation and dried by the vacuum oven.

### 3.3. Preparation of Pt/SiC Catalysts

A total of 50 mg of SiC powder was dispersed in the solution (consisting of 90 mL H_2_O, and 10 mL ethylene glycol). After 30 min of sonication, H_2_PtCl_6_ (3 mL, 10 mM) was added to the solution. The mixture was sonicated for another 30 min, and then sealed in a Teflon container at 120 °C for 180 min. The samples were separated by centrifugation and washed with ethanol and deionized water to remove any impurities. The final Pt/SiC (metallic Pt) was oven-dried overnight at 80 °C and stored for the subsequent experiments.

Aberration-corrected high-angle annular dark-field scanning transmission electron microscopy (AC-HAADF-STEM): The AC-HAADF-STEM images were collected with a Hitachi 2700C at the Center for Functional Nanomaterials at Brookhaven National Laboratory, which has a dedicated scanning transmission electron microscope (STEM), operating at 80 kV, 120 kV, and 200 kV, with a probe aberration-corrector to improve the imaging resolution to less than 1 Å.

### 3.4. X-ray Absorption Fine Structure (XAFS)

The XAFS spectra at Pt L_3_-edge for our samples were meticulously acquired from Beamline 8-ID within the esteemed confines of the National Synchrotron Light Source II, situated at Brookhaven National Laboratory. To ensure precision and accuracy, a Si (111) double crystal monochromator was employed to select the output beam. The energy calibration process was executed with the utmost care, utilizing a Pt foil as the reference standard. Our samples, handled with great attention to detail, were diligently pressed into pellets and hermetically sealed within Kapton films, a necessary step in preparing them for the ensuing XAFS measurements. Data acquisition took place at room temperature, employing the fluorescence mode and benefiting from the capabilities of a passivated implanted planar silicon (PIPS) detector, thus ensuring the highest quality of data. All XAFS results were processed using Demeter XAFS analysis software (0.9.26). The fitting process was performed following the procedures previously established [34]. Athena software (0.9.26) was applied for XAS data processing, including the conversion of raw data to μ(E) spectra, background subtraction and normalization, and Fourier transformation and plotting. Artemis software (0.9.26) was used for the analysis of extended X-ray absorption fine structure (EXAFS) data using theoretical standards, including setting the range of the Fourier transform from *k*-space and fitting range parameters in *R*-space. Reference samples of Pt foil and commercially acquired PtO_2_ were used for calibrating S_0_^2^ parameters.

### 3.5. X-ray Photoelectron Spectroscopy (XPS)

XPS were performed to characterize the variations of oxidation states of the Pt_1_/SiC. XPS analysis was conducted with a Versa Probe II Scanning XPS Microprobe (Physical Electronics (PHI), Chanhassen, MN, USA). For each sample, a survey analysis across the entire energy range and higher resolution analyses in the Pt 4f, Si 2p, and O 1s regions were performed.

### 3.6. Inductively Coupled Plasma Mass Spectrometry (ICP-MS) Experiments

ICP-MS was performed using a PerkinELmer SCIEX Elan DRC-e (PerkinELmer, Shelton, CT, USA)to determine the loading of Pt on SiC. The sample (10 mg) was acid-digested in mixtures of 7.5 mL 37% HCl, 2.5 mL 70% HNO_3_, and 2.0 mL 47.5% HF solutions using a Mars5 CEM microwave system (CEM corporation, Charlotte, NC, USA; first step, heating to 220 °C for 15 min; and second step, holding at 220 °C for 40 min). The samples were allowed to cool to room temperature for at least 3 h. To neutralize HF, 1.2 g of boric acid (ACS grade) was added to each solution. These solutions were then digested for a second time in the microwave oven. Each digested solution was then transferred into a polypropylene centrifuge tube and diluted to 50 mL with ultrapure water. From this solution, an aliquot of 0.15 mL was taken and diluted to 12 mL prior to ICP-MS analysis. According to our ICP-MS results, we determined the loading amount of single-atom Pt and Pt nanoparticle are 6.4 wt% and 10.3 wt%, respectively.

### 3.7. Temperature-Programmed Reduction (TPR)

TPR was used to evaluate the reduction conditions and thermal stability of the solid catalysts in a reductive environment. Gas flow rates were regulated using mass flow controllers, while the temperature was controlled using K-type thermocouples. This experiment involved loading 50 mg of Pt_1_/SiC catalyst into a straight tube quartz reactor containing a rough silica bed. The reactor was heated gradually from 100 °C to 900 °C at a rate of 10 °C/min. This temperature range was selected to investigate the optimal operating temperature for the pre-treatment process. During this phase, the hydrogen weight hourly space velocity (WHSV) was maintained at 12,000 mL g^−1^ h^−1^. A total gas flow rate of 100 mL/min was continuously purged during the reaction, with the feed gas composition consisting of 10% H_2_ and 90% Ar. Gas composition at the reactor outlet was monitored using an Standard Research Systems (SRS) RGA 100 Mass Spectrometer (Stanford Research Systems, Sunnyvale, CA, USA).

### 3.8. RWGS Reactions

A total of 50 mg of Pt_1_/SiC catalyst was loaded into a straight tube quartz reactor and heated from 100–900 °C at 10 °C·min^−1^. The total gas flow rate was 100 mL·min^−1^ and the inlet feed compositions were 20% H_2_ (WHSV = 24,000 mL g^−1^ h^−1^), 20% CO_2_, and 60% Ar. To quantify the CO_2_/H_2_ conversion, the outlet gas composition was compared to the measurements of the pre-reaction steady state CO_2_/H_2_ mass spectrometer signal.

## 4. Conclusions

In this work, we have developed a facile synthetic method to obtain the Pt_1_/SiC catalyst, which exhibits a superior catalytic performance surpassing the traditional Pt nanoparticle counterparts. The Pt_1_/SiC catalyst achieved an impressive conversion efficiency of approximately 54% at 900 °C, while displaying a high selectivity of up to 100% towards CO, as evidenced by the absence of other hydrocarbon byproducts. Moreover, the as-synthesized Pt_1_/SiC demonstrated remarkable thermal stability under a reductive H_2_ atmosphere. We additionally calculated the apparent activation barrier of the RWGS on Pt_1_/SiC to be 61.6 ± 6.4 kJ/mol, which is lower than most Pt-based catalyst reported to date. This finding highlights the exceptional catalytic performance of the Pt_1_/SiC catalyst and its potential for various industrial chemical processes, and provides pivotal inspirations for the rational design and optimization of SACs.

## Figures and Tables

**Table 1:** Best-fit parameters extracted from the Pt L_3_-edge FT EXAFS spectra of PtO_2_, Pt foil and Pt_1_/SiC. CN are coordination numbers for first coordination (Pt-O). R refers to the average interatomic distances between Pt and O atoms. σ^2^ are Debye Waller factors, revealing the variance of the distance distribution.

Sample	Shell	CN	R/Å	σ^2^/Å^2^
PtO_2_	Pt-O	6	2.06	0.0010 ± 0.0016
Pt foil	Pt-Pt	12	2.80	0.0013 ± 0.0014
Pt_1_/SiC	Pt-O	5.2 ± 0.7	2.05 ± 0.01	0.0051 ± 0.0011

## Data Availability

Not applicable.

## Supplementary Materials

The following supporting information can be downloaded at https://www.mdpi.com/article/10.3390/molecules28186630/s1: Section S1: Turnover Frequencies (TOF) Calculation. Section S2: Calculation of the Activation Energy. Figure S1: Binding energy of O 1s for pristine SiC by high-resolution XPS spectra. O peak position at 532.6 eV demonstrate the surface -OH groups, which are critical to the stabilization of Pt_1_; Figure S2: setup for TPR and RWGS experiments; Figure S3: (a,b) High-resolution transmission electron microscopy (HRTEM) images, and (c) energy dispersive spectroscopy (EDS) of Pt/SiC (metallic Pt), synthesized via hydrothermal method with ethylene glycol as the reducing agent; Figure S4: zeta potential of pristine SiC vs. pH value; Table S1: comparisons of catalysts towards RWGS reaction. References [35,36,37,38,39,40,41,42] are cited in the Supplementary Materials.
