# Supplementary material for: Single-Atom Platinum Catalyst for Efficient CO2 Conversion via Reverse Water Gas Shift Reaction
Source: Molecules. 2023 Sep 14;28(18):6630. doi: 10.3390/molecules28186630 (PMC10534439; doi:10.3390/molecules28186630)
Supplement: Supplementary file 1 [file molecules-28-06630-s001.zip › molecules-2612726-supplementary.pdf]

Supplementary Materials

Single-Atom Platinum Catalyst for Efficient CO_2 Conversion via Reverse Water Gas Shift Reaction

Yulian He [1] and Dahong Huang [2,*]

[1] Shanghai Jiao Tong University Joint Institute, University of Michigan, Shanghai 200240, China; yulian.he@sjtu.edu.cn
[2] CAS Key Laboratory of Urban Pollutant Conversion, Department of Environmental Science and Engineering, University of Science and Technology of China, Hefei 230026, China
* Correspondence: dahonghuang@ustc.edu.cn

Section S1. Turnover Frequencies (TOF) Calculation

TOF indicates the amount of converted CO_2 on per mol of Pt atoms in one second. TOF of Pt_1/SiC and metallic Pt/SiC at 900°C were calculated as follows:

$$\text{TOF } (Pt_1/SiC) = \frac{54\% \times 20\ mL/(22.4\frac{L}{mol} \times 60\ s)}{6.4\ wt\% \times 0.05\ g\ /195\ (g/mol)}$$

$$= 0.49\ mol_{co2}\ mol_{Pt}^{-1}\ S^{-1}$$

Section S2. Calculation of the Activation Energy

The Arrhenius is a graphical representation of the natural logarithm of kinetic constants, ln(k), plotted against the inverse of temperature (1/T). This plot follows the Arrhenius equation, which was originally proposed by Svante Arrhenius in 1889 and now is extensively used to determine the activation energy of a reaction. The correlation between ln(k) and 1/T can be expressed as follows:

$$ln\frac{k_2}{k_1} = \frac{E_a}{R}(\frac{1}{T_1} - \frac{1}{T_2})$$

Where k represents the reaction rate, E_a is the activation energy, R is the ideal gas constant (8.314 J mol^{-1} K^{-1}) and T stands for the temperature in Kelvin.

By plotting ln(k) against 1/T, the slope of the resulting line can be used to calculate the E_a of reaction using specific catalyst, allowing the investigation of the temperature dependence of a reaction based on experimental data and thus the evaluation of catalytic performance of catalysts.

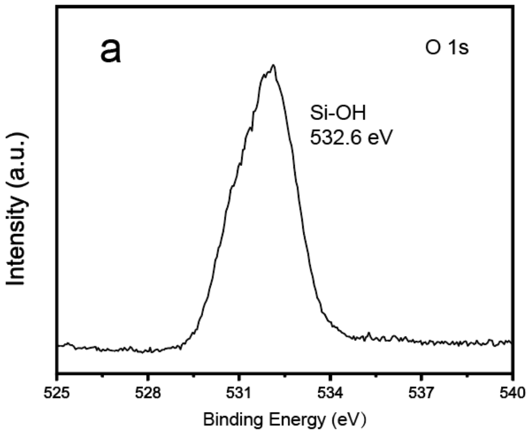

Figure S1. Binding energy of O 1s for pristine SiC by high-resolution XPS spectra. O peak position at 532.6 eV demonstrate the surface -OH groups, which are critical to the stabilization of Pt_1.

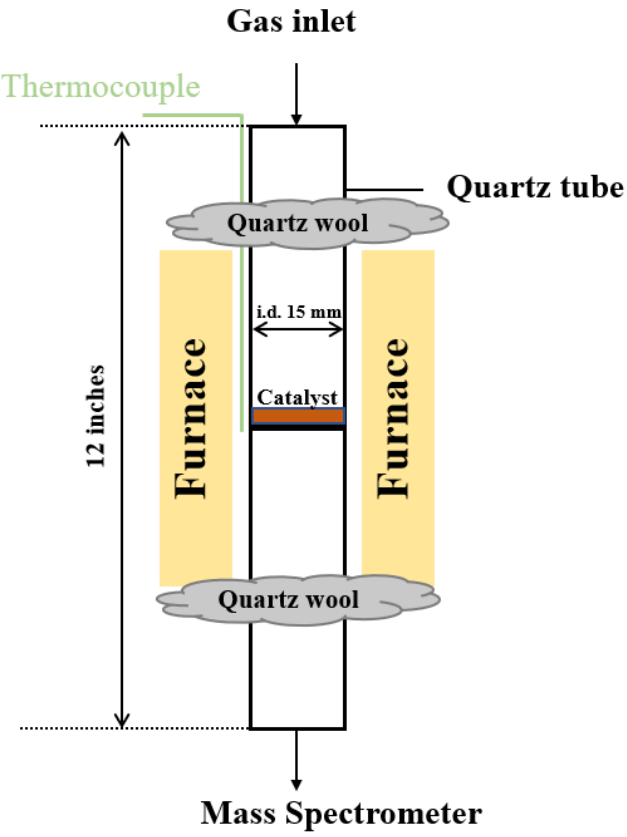

Figure S2. Setup for TPR and RWGS experiments.

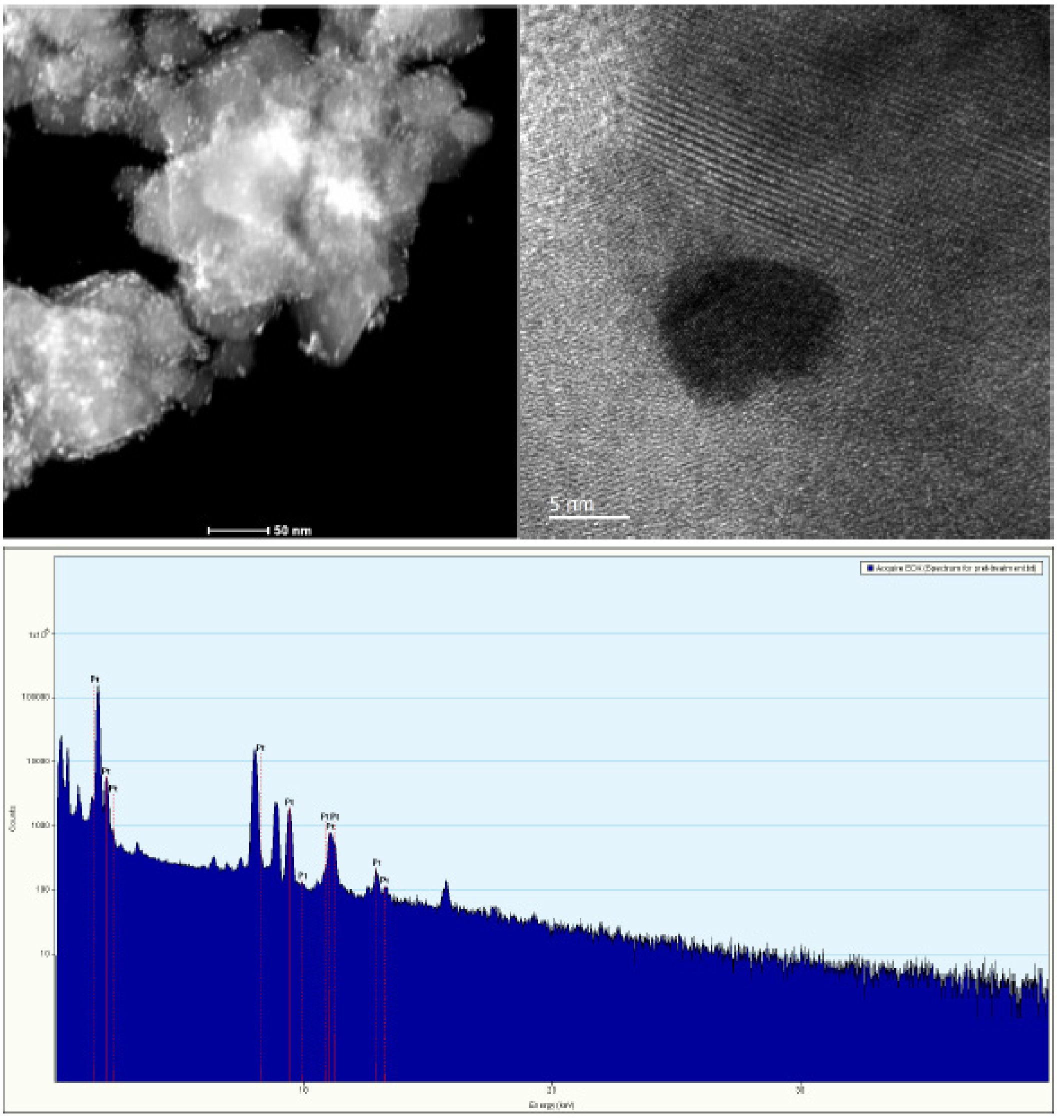

Figure S3. (a, b) High-resolution transmission electron microscopy (HRTEM) images and (c) energy dispersive spectroscopy (EDS) of Pt/SiC (metallic Pt), synthesized via hydrothermal method with ethylene glycol as the reducing agent. The EDS shows successful loading of Pt nanoparticle onto the SiC substrate.

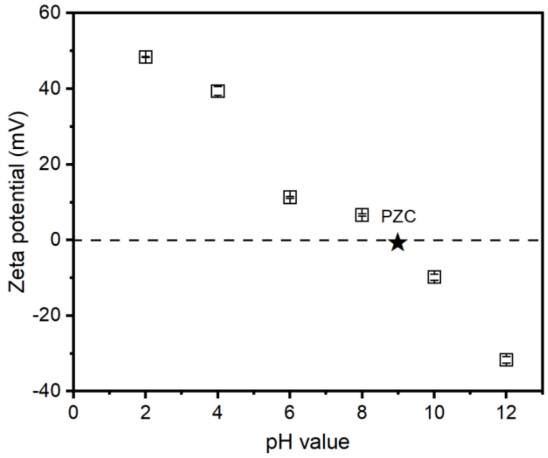

Figure S4. Zeta potential of pristine SiC *vs* pH value.

Table S1. Comparisons of catalysts towards RWGS reaction.

Catalyst	Conversion (%)	Selectivity (%)	Temperature (°C)	H_2:CO_2	Activation energy (kJ/mol)	Reference
Pt_1/SiC	54	100	900	1:1	61.6 ± 6.4	This work
Pt/CeO_2	30	100	500	1:1	68	[36]
Pt/ZSM-5	27.4	95	500	1:1	80 ± 5.1	[37]
NiO/CeO_2	50	100	700	1:1	65.65	[38]
Cu–Ni/ɣ-Al_2O_3	29	80	600	1:1	N/A	[39]
Cu/SiO_2	5.3	N/A	600	1:1	N/A	[40]
Cu/β-Mo_2C	40	100	600	2:1	N/A	[41]
Cu-ZnGaZrO	16.8	99.7	325	3:1	70.9 ± 3.7	[42]
CuFe/Al_2O_3	42	N/A	700	1:1	N/A	[43]
